# Supplementary figures and images for: TriPer, an optical probe tuned to the endoplasmic reticulum tracks changes in luminal H2O2
Source: BMC Biol. 2017 Mar 27;15:24. doi: 10.1186/s12915-017-0367-5 (PMC5368998; doi:10.1186/s12915-017-0367-5)

Fig. S1

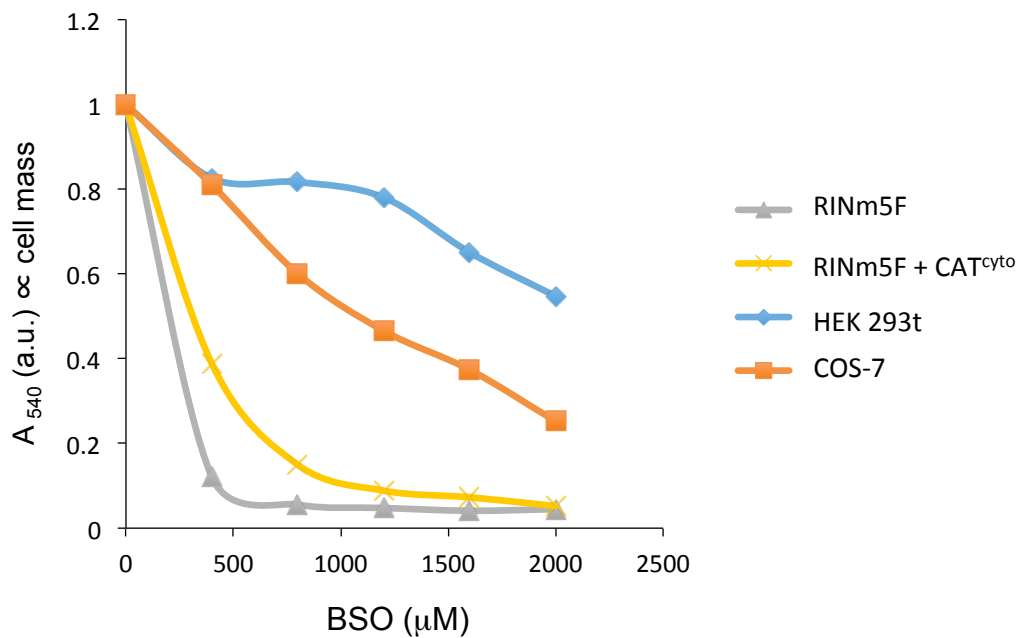

Supplement: Supplementary file 1 — Variable sensitivity of cultured cells to glutathione depletion. As in Fig. 1a, absorbance at 540 nm (an indicator of cell mass) by cultures of parental RINm5F, RINm5F stably overexpressing catalase in their cytosol (CATcyto), HEK293, or COS7 cells that had been exposed to the indicated concentration of BSO before fixation and staining with crystal violet. (PDF 91 kb) [file 12915_2017_367_MOESM1_ESM.pdf]

Fig. S2

A

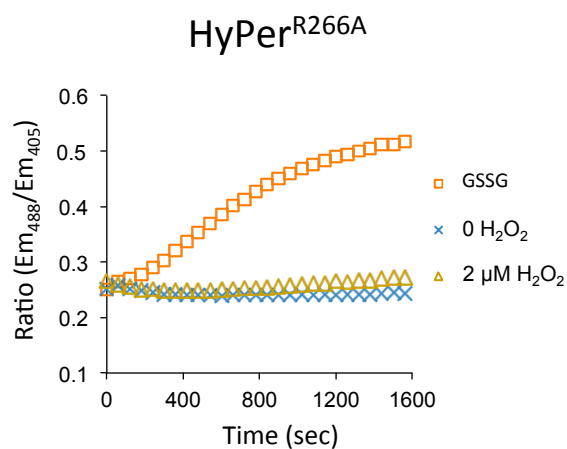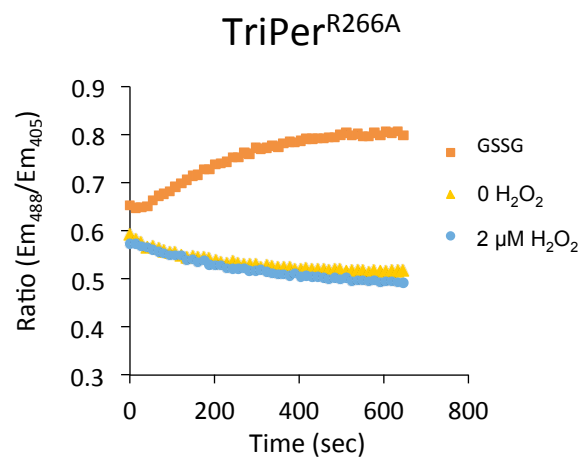

B

**TriPer<sup>R266A</sup>**

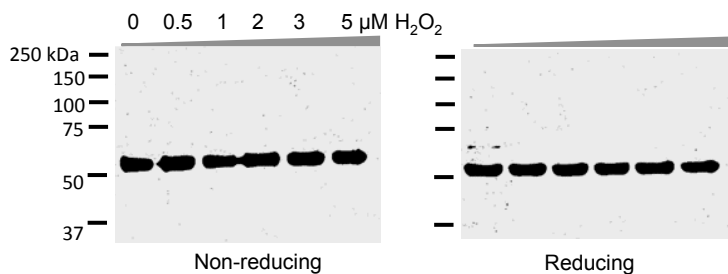

C

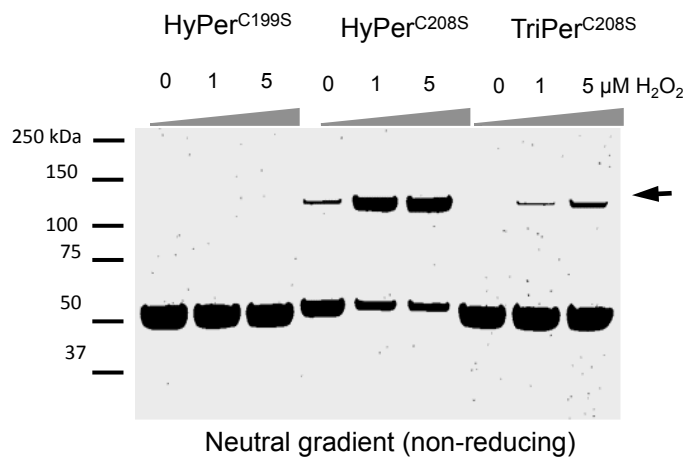

D

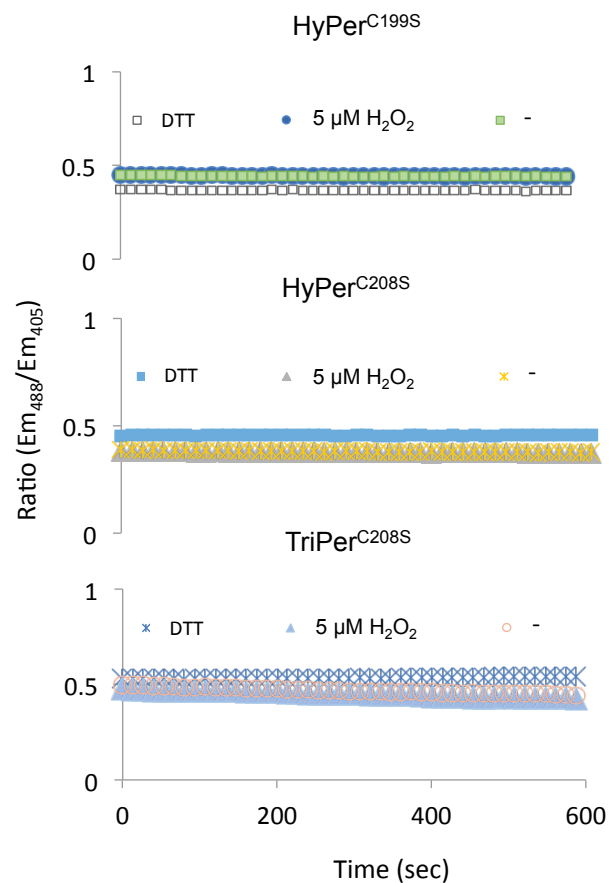

Supplement: Supplementary file 2 — The role of R266 in HyPer and TriPer’s reactivity with H2O2. (A) Traces of time-dependent changes to the excitation ratio of recombinant HyPer or TriPer variants with the inactivating R266A mutation. (B) Non-reducing and reducing SDS-PAGE of recombinant TriPerR199A following incubation with increasing concentrations of H2O2 for 15 min. (C) Non-reducing gradient SDS-PAGE (pH 7.3, 4–12%) of samples as in Fig. 2g. (D) Traces of time-dependent changes to the excitation ratio of HyPer and TryPer mutant variants treated as in (B). Note that the variants lacking the ability to form C199-C208 disulfide do not change their excitation ratio upon oxidation. (PDF 93 kb) [file 12915_2017_367_MOESM2_ESM.pdf]

**Fig. S3**

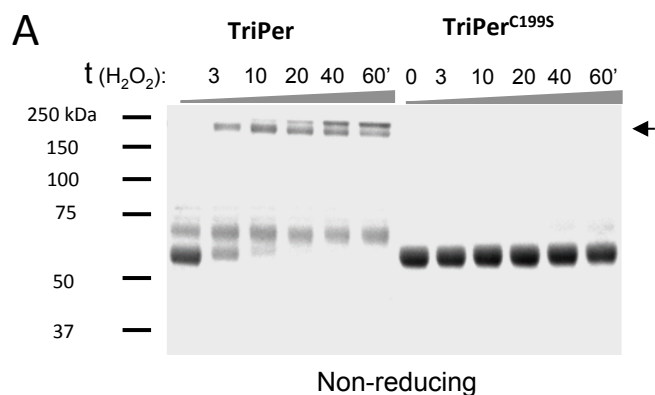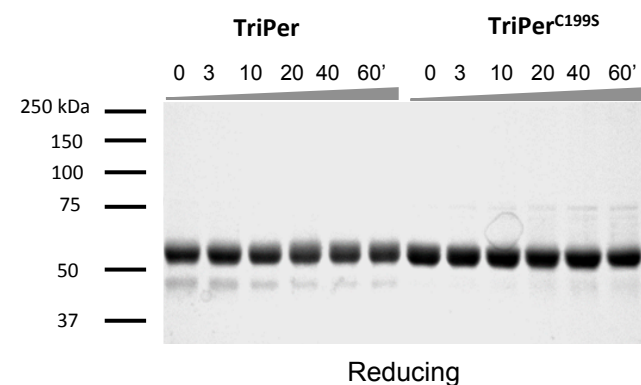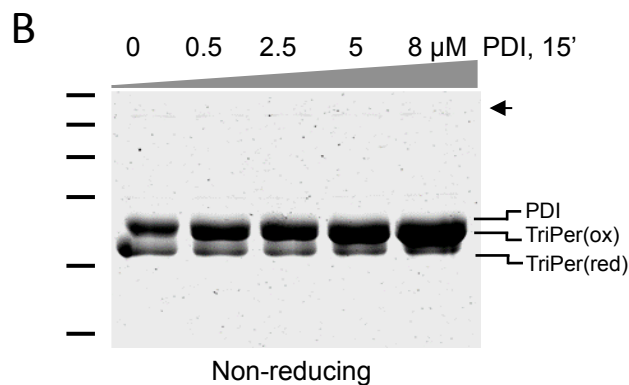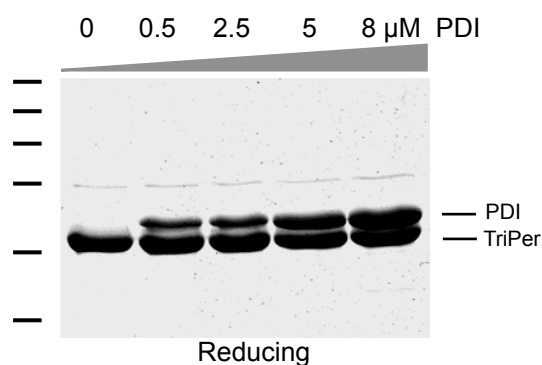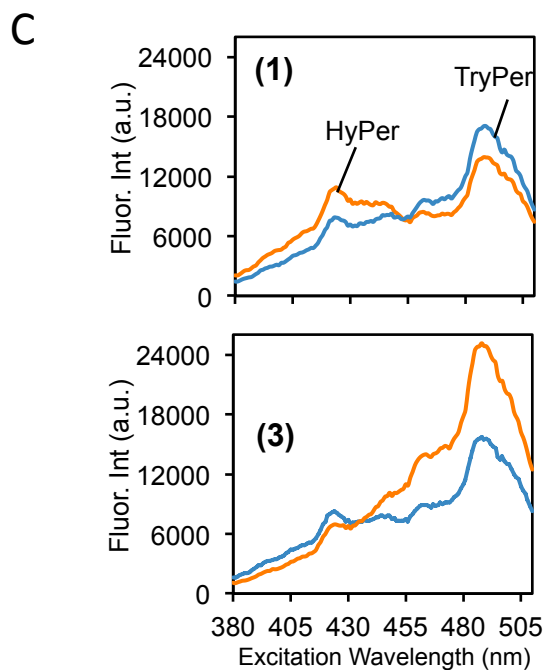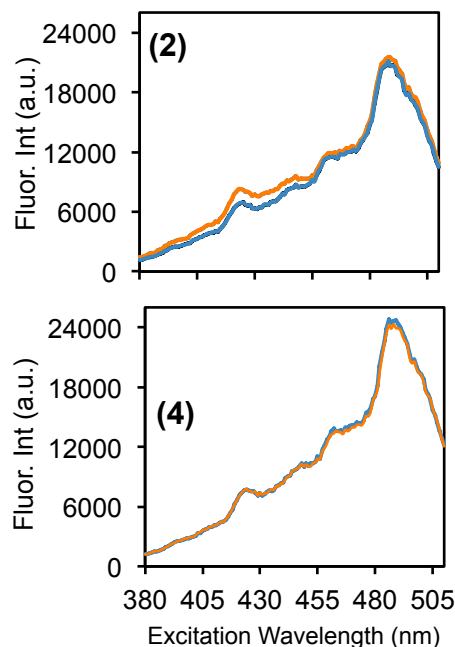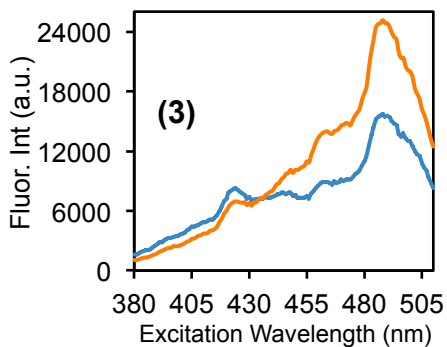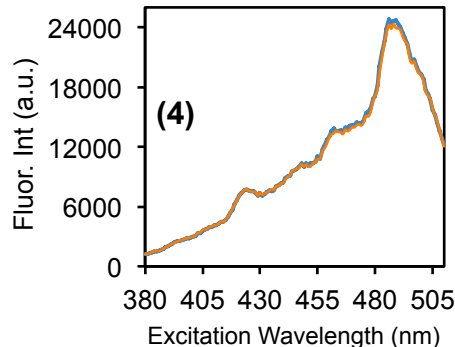

Supplement: Supplementary file 3 — In vitro, H2O2-driven formation of disulfide-bonded high molecular weight TriPer species with divergent optical properties. (A) Coomassie-stained non-reducing and reducing SDS-PAGE of wild-type TriPer or its mutant variant lacking the peroxidatic cysteine (TriPer C199S) following exposure to 1.5 μM of H2O2 for the indicated time period. Black arrow denotes disulfide-bonded high molecular weight TriPer species. (B) As in (A), but following a 15-min exposure to increasing concentrations of oxidized PDI (0–8 mM). Note the lack of the high molecular weight species in this sample and their prominence in the H2O2-treated sample, (A) above. (C) Excitation spectra (measured at emission 535 nm) of HyPer (orange trace) and TriPer (blue trace) for the different states of the probes, corresponding to phases 1–4 in Fig. 3c. (PDF 205 kb) [file 12915_2017_367_MOESM3_ESM.pdf]

Fig. S4

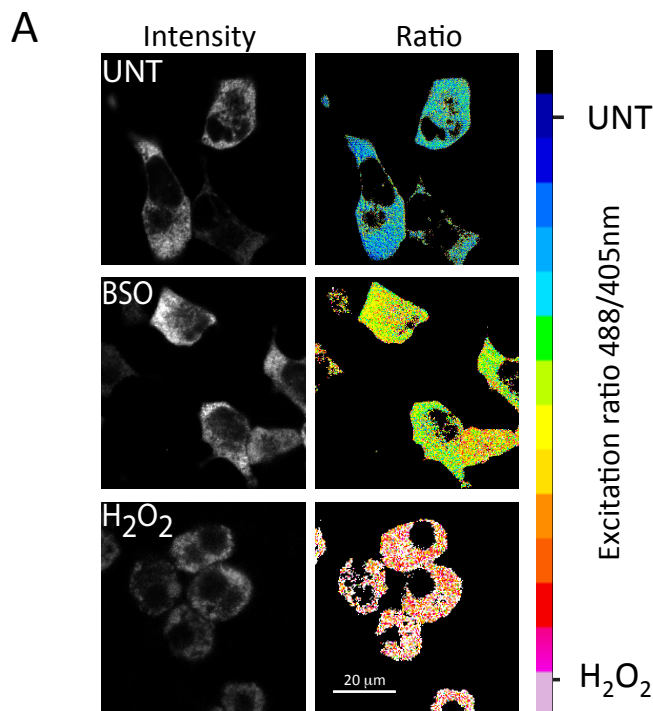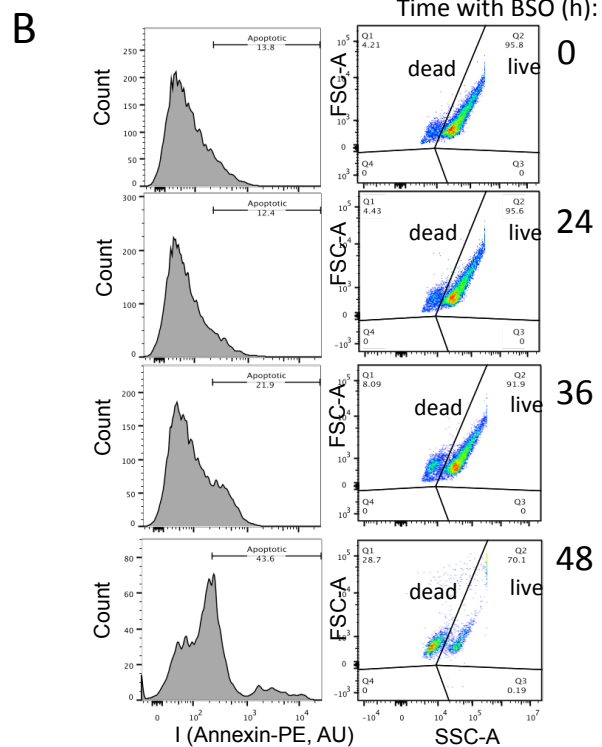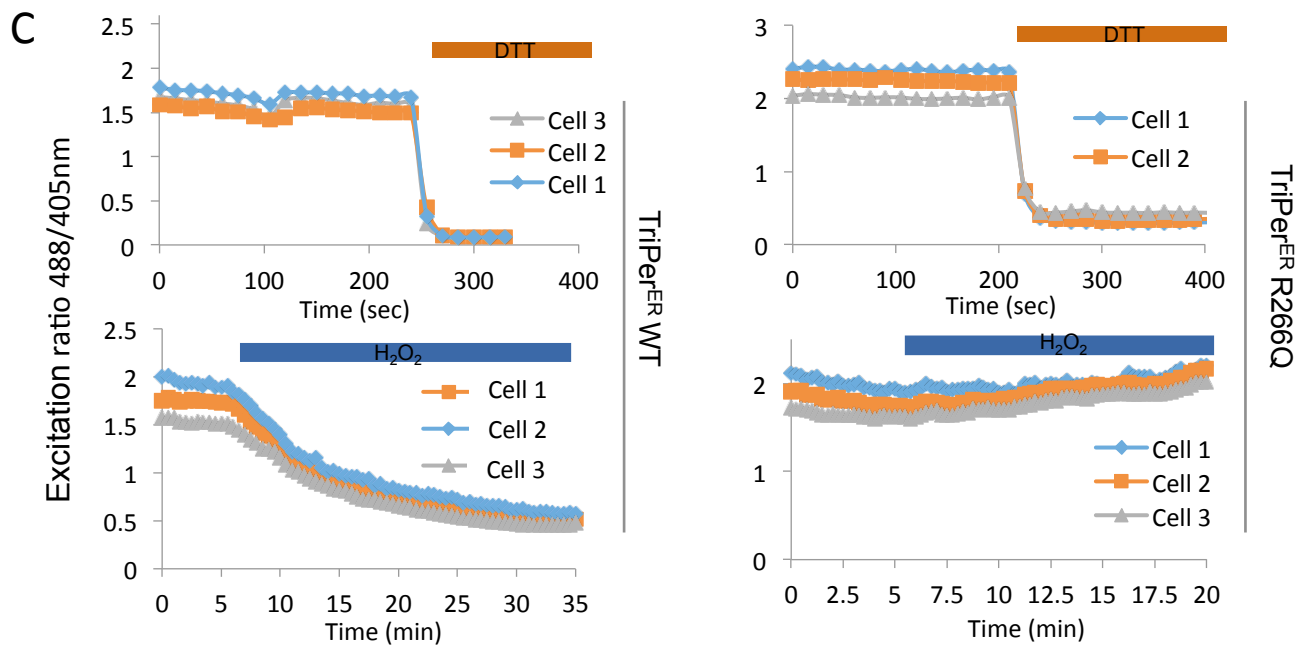

Supplement: Supplementary file 4 — Glutathione depletion induced apoptotic cell death and leads to concordant changes in TriPerER optical properties. (A) Photomicrographs and fluorescence excitation ratiometric images of untreated (UNT) RINm5F cells transiently expressing TriPerER or cells treated with BSO (0.3 mM, 28 h) or H2O2 (0.2 mM, 15 min). The images were color coded for 488/405 nm excitation ratio (R488/405) according to the color map shown. (B) Flow cytometry analysis of RINm5F cells at the indicated time points after exposure to BSO (0.3 mM). Populations of dead and live cells were resolved by plotting forward vs. side scattering amplitudes (FCS-A and SSC-A accordingly). Apoptotic cell populations were assessed by detecting surface phosphatidylserine using phycoerythrin (PE) conjugated Annexin V. Note that a significant population of dead cells only emerges after 36 h, whereas an increase in the ER H2O2 signal is observed by 12 h (Fig. 6c). (C) A ratiometric trace of TriPerER WT or TriPerER containing an R266Q mutation expressed in RINm5F cells, exposed to H2O2 (0.2 mM) or DTT (2 mM) for the indicated duration. (PDF 636 kb) [file 12915_2017_367_MOESM4_ESM.pdf]
