# Supplementary material for: TriPer, an optical probe tuned to the endoplasmic reticulum tracks changes in luminal H2O2
Source: BMC Biol. 2017 Mar 27;15:24. doi: 10.1186/s12915-017-0367-5 (PMC5368998; doi:10.1186/s12915-017-0367-5)
Supplement: Supplementary file 5 — List of plasmids. (PDF 56 kb) [file 12915_2017_367_MOESM5_ESM.pdf]

Table S1. List of plasmids

ID*	Plasmid name	Description	Reference	First appearance	Label in figure
1820	pLVX_TRE3G_hINS	Mammalian expression of tetracyclin inducilbe human proinsulin	This study	Figure 1	Tet-Ins
1824	pLVX-EF1a-Tet3G	Regulator plasmid for Tet-On® 3G Inducible Expression System	Clontech	Figure 1	Tet-Ins
816	HyPer_pSmt3_pET28b	Bacterial expression of HyPer	PMID: 26504166	Figure 2	HyPer
1046	TriPer_pSmt3_pet28b(MP2)	Bacterial expression of TriPer**	This study	Figure 2	TriPer
1045	HyPerC199S_pSmt3_pET28b	Bacterial expression of HyPer lacking peroxidaic cys, C199S	This study	Figure 2	HyPerC199S
1057	TriPer_C199S_pSmt3_pet28b (MP2)	Bacterial expression of TriPer lacking peroxidaic cys, C199S	This study	Figure 2	TriPer C199S
779	HyPerC208S_pSmt3_pQE30	Bacterial expression of HyPer lacking resolving cys, C208S	This study	Figure 2	HyPer C208S
1905	TriPerC208S_pSmt3_pET28b	Bacterial expression of TriPer lacking resolving cys, C208S	This study	Figure 2	TriPer C208S
827	HyPerR266A_pSmt3_pET28b	Bacterial expression of HyPer lacking peroxidaic R266	This study	Figure S2	HyPer R266A
1909	TriPerR266A_pSmt3_pET28b	Bacterial expression of TriPer lacking peroxidaic R266	This study	Figure S2	TriPer R266A
233	hPDI(18-508)pTrcHis-A	Bacterial expression of human PDI1A	PMID: 21145486	Figure 3	PDI
887	hPDI_WT_mCherry_KDEL-N3 (MP#1)	Mammalian expression of human PDI1A C-term fused to mCherry	PMID: 25575667	Figure 4	PDI-mCherry
601	pFLAG_ERHyPer_CMV1	Mammalian expression of ER HyPer	PMID: 26504166	Figure 4	HyPerER
1025	pFLAG_ERTriPer_CMV1	Mammalian expression of ER TriPer	This study	Figure 4	TriPerER
777	pHyPer_cyto	Mammalian expression of cytoplasmic HyPer	PMID: 20692175	Figure 5	cytoHyPer
1907	pFLAG_ERTriPer R266Q_CMV1	Mammalian expression of ER TriPer lacking peroxidaic R266	This study	Figure S4	TriPer R266Q
527	FLAGM1_roGFP_iE_pCDNA3	Mammalian expression of ERroGFPiE	PMID: 23589496	Figure 6	ERroGFPiE
988	FLAGM1_mChaC1_CtoS_mCherry_pCDNA5_FRT_TO	Mammalian expression of ER ChaC1 C-term fused to mCherry	PMID: 25073928	Figure 6	ChaC1 wt
1028	FLAGM1_mChaC1_CtoS_E116Q_mCherry_pCDNA5	Mammalian expression of inactive ER ChaC1 C-term fused to mCherry	PMID: 25073928	Figure 6	ChaC1 mut

*Unique plasmid identification number used internally (a lab number).

** Cysteine was introduced instead of alanine 187, which is found in vicinity fo C208 in the OxyR structure PDB1I69, PMID: 11301006; amino asid numbering as in OxyR
